# Enhancing Clinical Data Infrastructure for AI Research: Comparative Evaluation of Data Management Architectures

**DOI:** 10.2196/74976

**Published:** 2025-08-01

**Authors:** Richard Gebler, Ines Reinecke, Martin Sedlmayr, Miriam Goldammer

**Affiliations:** 1 Faculty of Medicine and University Hospital Carl Gustav Carus, Dresden University of Technology Dresden Germany; 2 Data Integration Center, Center for Medical Informatics, University Hospital Carl Gustav Carus at Dresden University of Technology Dresden Germany

**Keywords:** clinical data management, big data in health care, data architecture evaluation, data warehouse, data lake, data lakehouse

## Abstract

**Background:**

The rapid growth of clinical data, driven by digital technologies and high-resolution sensors, presents significant challenges for health care organizations aiming to support advanced artificial intelligence research and improve patient care. Traditional data management approaches may struggle to handle the large, diverse, and rapidly updating datasets prevalent in modern clinical environments.

**Objective:**

This study aimed to compare 3 clinical data management architectures—clinical data warehouses, clinical data lakes, and clinical data lakehouses—by analyzing their performance using the FAIR (findable, accessible, interoperable, and reusable) principles and the big data 5 V’s (volume, variety, velocity, veracity, and value). The aim was to provide guidance on selecting an architecture that balances robust data governance with the flexibility required for advanced analytics.

**Methods:**

We developed a comprehensive analysis framework that integrates aspects of data governance with technical performance criteria. A rapid literature review was conducted to synthesize evidence from multiple studies, focusing on how each architecture manages large, heterogeneous, and dynamically updating clinical data. The review assessed key dimensions such as scalability, real-time processing capabilities, metadata consistency, and the technical expertise required for implementation and maintenance.

**Results:**

The results show that clinical data warehouses offer strong data governance, stability, and structured reporting, making them well suited for environments that require strict compliance and reliable analysis. However, they are limited in terms of real-time processing and scalability. In contrast, clinical data lakes offer greater flexibility and cost-effective scalability for managing heterogeneous data types, although they may suffer from inconsistent metadata management and challenges in maintaining data quality. Clinical data lakehouses combine the strengths of both approaches by supporting real-time data ingestion and structured querying; however, their hybrid nature requires high technical expertise and involves complex integration efforts.

**Conclusions:**

The optimal data management architecture for clinical applications depends on an organization’s specific needs, available resources, and strategic goals. Health care institutions need to weigh the trade-offs between robust data governance, operational flexibility, and scalability to build future-proof infrastructures that support both clinical operations and artificial intelligence research. Further research should focus on simplifying the complexity of hybrid models and improving the integration of clinical standards to improve overall system reliability and ease of implementation.

## Introduction

As digital technology advances, health care organizations are confronted with even larger volume and more varied datasets produced by modern sensors and wearables, the adoption of electronic health records, and advancements in medical imaging and genome sequencing [1-3].

The current growth in artificial intelligence (AI) applications, including predictive analytics and personalized medicine systems to multimodal diagnostic decision-support systems, has revealed requirements for data and the underlying data management architectures [4]. These include the following:

- High-quality, bias-controlled, and representative training data to avoid “garbage in, garbage out” and provide reliable outputs [5]
- Transparency through provenance, versioning, and integrity to ensure reproducible and auditable results [5]
- Standards-based interoperability to ensure data content and exchange [6]
- Ability to handle large sized, multimodal datasets [5]

These requirements align closely with 2 widely accepted approaches to evaluate data management solutions. Data quality, provenance, accessibility, and reuse correspond to the FAIR (findable, accessible, interoperable, and reusable) principles, while size and multimodal properties align with the 5 V’s (volume, variety, velocity, veracity, and value) of big data. Value reflects the combined benefit to downstream analytics of these 5 V’s. Adopting this combined FAIR-5V perspective on AI life cycle data-needs enables us to avoid creating yet another requirements checklist while still capturing bias control, transparency, and real-time capability.

The traditional clinical data warehouse (cDWH), a data architecture that has been established for decades [7], was designed as a central data hub. It harmonizes data from various sources, optimizes data processing by ensuring interoperability standards, and protects patient data, which are organized in table structures to create one reliable source of truth for all data storage systems. In DWHs, transactions follow atomicity, consistency, isolation, and durability (ACID) properties. Atomicity means transactions are all-or-nothing, consistency ensures data remains the same before and after transactions, isolation means transactions do not affect each other, and durability ensures that once a transaction is completed, it stays saved. If there is a failure or a change in the data structure, the existing data are not affected. In addition, changes are not made automatically. These properties ensure the data are valid and reflect the high priority of data validity in health care, enabling retrospective quality audits and compliance reporting.

However, cDWHs now face growing challenges. The rapid increase in data volume from diverse sources, such as imaging, sensor data, and genomics, makes it difficult to maintain the traditional fixed schema approach, preventing a unified patients view containing continuous or unstructured data [8]. In addition, AI-driven workflows require not only large and varied datasets but also near real-time data processing to handle streaming information and provide timely insights [9]. With weak support of real-time data, these cDWHs delay detection of acute events reducing the window for early inventions. If data arrives in formats with inconsistent structures or with missing values, it can reduce the accuracy and reliability of AI outputs. This situation has led to a new set of technical demands on data management, including more flexible storage, advanced data governance strategies, and scalable infrastructure for large-scale analytics [10].

To meet these increasing requirements, more recent concepts of data lakes and data lakehouses have been adapted to the clinical context, resulting in clinical data lakes (cDLs) and clinical data lakehouses (cDLHs).

A cDL is an architectural approach that enables the storage of large amounts of raw data in its original format, including structured, semistructured, and unstructured data [11]. This is in contrast to traditional cDWHs, which store data in a highly structured, organized way. cDLs are designed to provide high scalability and flexibility in data storage, allowing organizations to store data without the need for a defined schema first. This enables multimodal views on patients using diverse datasets, including real-time processing, but comes with drawbacks in data governance and data quality challenges for clinical decision-making.

The cDLH is a new, more complex hybrid approach that combines the scalable storage options of a cDL with the structured queries and performance optimizations of a cDWH [12]. This architecture aims to overcome the limitations of the two previous models by providing a platform on which raw data can be stored cost-effectively and processed in structured formats as required by applying open data formats and ACID transactions as well as comprehensive data management techniques. However, this architecture is still relatively new, highly complex, and designed primarily for cloud-based infrastructures, which may not align with the strict on-premise requirements of many European health care institutions.

This paper analyses and compares these three data management architectures—cDWH, cDL, and cDLH—using established technical and data governance requirements—guided by the FAIR principles and the 5 V’s of big data. The aim of this paper is to develop practical guidance for health care institutions of different aims and sizes, offering support in choosing a future-oriented data architecture that improves both medical research and patient care and promotes the adoption of AI.

In the following sections, we describe our methodology, present the comparative results, discuss the implications of our findings, and conclude with recommendations.

## Methods

### Overview

To perform a systematic analysis and comparison of data management architectures designed to manage clinical data, we applied a comprehensive methodological framework that integrates the 5 V’s of big data with the FAIR principles. The resulting requirements are specifically tailored to the needs of big data research in health care to enable the comparison of the data architectures.

### Step 1: Defining Analysis Criteria

To enable a comprehensive comparison of the architectures for clinical data management, we decided to analyze 2 different types of requirements. On the one hand, we aimed to analyze aspects that relate to the more fundamental handling and management of the data itself and can be summarized under the term data governance. The FAIR principles are a suitable framework for this, which has been established in recent years [13,14]. By contrast, we aim to cover technical requirements, for example, the handling of large amounts of data or the speed of data updates. These are considered by deriving analysis criteria from the 5 V’s of big data. We then adapted both frameworks to the clinical context and specified their general definitions to highlight health care characteristics, for example, interoperability standards in health care and typical complexity of clinical data. Moreover, we emphasize certain aspects that are directly relevant to AI applications, such as data quality and real-time processing. This underlines the importance of having a data architecture that can support advanced AI methods.

### Step 2: Conducting a Rapid Literature Review

On the basis of the requirements defined in section 2.1, a rapid literature review was conducted in relevant databases on the PubMed, Scopus, ACM Digital Library, and Web of Science databases.

We focused on literature that addressed the relationship of data architectures to the FAIR principles or to at least 3 of the 5 V’s of big data (volume, velocity, variety, veracity, and value).

We applied inclusion and exclusion criteria as shown in Textbox 1.

**Inclusion and exclusion criteria for the rapid review.**

**Inclusion criteria**

- Discussed at least 1 of the data architectures (clinical data warehouses, clinical data lakes, or clinical data lakehouses)
- Addressed the FAIR principles (findable, accessible, interoperable, and reusable) or covered at least 3 of the 5 V’s (volume, variety, velocity, veracity, and value) of big data in a meaningful way
- Connected these frameworks (FAIR or 5 V’s) with the chosen data architecture
- Focused on clinical, health care, or biomedical contexts

**Exclusion criteria**

- Was not available as a full text
- Was not written in English

Following a PRISMA (Preferred Reporting Items for Systematic reviews and Meta-Analyses)-like workflow, all records from the 4 databases were assembled using the combined search string, as provided in Textbox 2.

**Search string for the literature search.**

(“clinical” OR “medical” OR “healthcare” OR “biomedical”) AND (“lakehouse” OR “data lake” OR “data warehouse”) AND (“FAIR Principles” OR (“findable” AND “accessible” AND “interoperable” AND “reusable”) OR “5V” OR ((“volume” AND “velocity” AND “variety”) OR (“volume” AND “velocity” AND “veracity”) OR (“volume” AND “velocity” AND “value”) OR (“volume” AND “variety” AND “veracity”) OR (“volume” AND “variety” AND “value”) OR (“volume” AND “veracity” AND “value”) OR (“velocity” AND “variety” AND “veracity”) OR (“velocity” AND “variety” AND “value”) OR (“velocity” AND “veracity” AND “value”) OR (“variety” AND “veracity” AND “value”)))

Next, duplicate records were removed, followed by a title and abstract screening to quickly exclude papers that were clearly out of scope. Finally, a full-text review of the remaining papers was performed to determine whether they met all inclusion criteria. As the review concentrated on technical architecture descriptions instead of clinical-effect estimates, conventional risk-of-bias instruments were not applicable. As a result, all 9 studies were treated as evidence based on narrative. During this process, 2 persons independently screened the titles, abstracts and full-paper.

Then, a detailed review of these studies and documentation of their key characteristics were carried out, resulting in a comparison table. We have chosen to present each criterion qualitatively. This is to prevent a single numeric score from hiding context-specific priorities.

### Step 3: Developing Recommendations for Health Care Data Management

On the basis of the results of the literature review and supplemented with secondary literature, we developed a set of recommendations. Given that AI applications rely heavily on the underlying data infrastructure, it is essential that the recommendations also ensure that data are optimized for advanced AI analytics. These recommendations are aimed at health care executives and technical experts involved in the evaluation, selection, and implementation of data management solutions. In addition, we considered the implications of each data architecture for AI applications. For example, the ability to support real-time data streaming, high-quality structured data, and flexible schema management are key for AI-driven tasks, such as clustering, classification, and predictive analytics. Detailed considerations on these recommendations will be presented in the Discussion section.

Therefore, in this paper, we present the complete process from requirements through comparison of variants to use-case-specific recommendations for clinical data architectures.

### Ethical Considerations

As no personal data were processed during this research, no ethical approval was necessary. All relevant data are referenced in this paper. Although not reviewed by an ethics committee, all procedures strictly followed ethical guidelines and adhered to the Declaration of Helsinki.

## Results

### Overview

To arrive at our findings, we first present a set of comprehensive requirements derived from existing frameworks. Given the increasing role of AI in health care, these requirements are critical as they support the data quality and structure that AI systems demand. These requirements serve as the basis for our following analysis of clinical data architectures.

### Defining Analysis Criteria

The FAIR principles set out the most important governance requirements for data management. Specified for (clinical) data architectures, these aspects ensure that the data are [15] findable, accessible, interoperable, and reusable, as described in the following sections.

#### Findable

Data must be identified by unique and persistent identifiers [15] (eg, globally unique identifiers). It should be indexed or cataloged so both humans and machines can locate relevant datasets easily. Descriptive metadata must be consistently provided [15]. While “findable” might intersect with “interoperable” in terms of using common standards, its main emphasis is on how easily data can be located rather than how data integrates with other systems. In a clinical context, patient data often resides in multiple departments (eg, the main clinical information system or specialized systems for laboratory results, and imaging data). Unique identifiers, such as the patient IDs, medical case IDs, and laboratory result IDs as well as well-structured metadata enable clinicians or researchers to quickly find and retrieve the needed information [14].

#### Accessible

Data should be retrievable via standard communication protocols and appropriate authentication or authorization methods [15]. Even if the data itself becomes unavailable over time, associated metadata should remain accessible [15]. “Accessible” emphasizes secure retrieval under clearly defined rules [15]. This differs from “findable” (which focuses on locating data) and from “interoperable” (which focuses on format and standardization). In a clinical context, a clinician who, for example, needs a patient’s magnetic resonance imaging scans from the radiology archive must be able to securely access the images through hospital-approved protocols (eg, Digital Imaging and Communications in Medicine servers and controlled hospital networks [13]). This ensures that sensitive data are protected yet still available to authorized personnel.

#### Interoperable

Data are stored in commonly applicable, machine-readable formats, using standardized vocabularies (eg, Systemized Nomenclature of Medicine–Clinical Terms. and Logical Observation Identifiers Names and Codes) to maintain semantic consistency. It should link to related datasets and metadata, enabling systems to exchange and understand the information [15]. “Interoperable” focuses on seamless data exchange and shared definitions. It does not overlap with “accessible,” which deals with how authorized users obtain data, or “reusable,” which deals with how data can be used repeatedly. In a clinical context, when patient data are transferred between hospitals or systems, interoperability ensures that, for example, terms such as “blood pressure” or “fever” are interpreted identically. Thus, a cardiology department in hospital A can share vital signs data with hospital B’s critical care unit without losing meaning, thanks to standardized formats such as Health Level7 Fast Healthcare Interoperability Resources [13].

#### Reusable

Data must have detailed domain-specific metadata and clear usage licenses or conditions so that it can be effortlessly repurposed for new studies or applications [15]. “Reusable” highlights the potential for data to be used in future projects. This does not repeat “findable” or “interoperable,” as it deals with the conditions enabling repeated use rather than how data are discovered or shared. In a clinical context, for example, a research group conducting a clinical trial on diabetes may decide to use past laboratory measurements (eg, glucose levels) if these values are documented in a standardized format. Clearly stated permissions and provenance records allow them to reuse the data responsibly, improving reproducibility and speeding up new investigations [14].

While the FAIR principles address data management and governance aspects, ensuring that data can be effectively found, retrieved, combined, and reused, the area of big data also presents significant technical challenges. These challenges are captured by the 5 V’s of big data—volume, velocity, variety, veracity, and value—which serve as essential characteristics and requirements for modern data management systems.

#### Volume

Volume addresses the challenge of storing, processing, and managing large datasets from sources [16,17] such as electronic health records, whole-genome sequencing, and high-resolution imaging. It includes handling structured clinical data and the costs of scaling storage and processing capabilities. This ensures data integrity and accessibility without sacrificing security. “Volume” purely addresses the size and storage challenges. It does not address different data formats (which are the focus of “variety”), or data flow speed (which is the focus of “velocity”).

#### Variety

Variety refers to the management of different types of medical data [16]. This includes structured data from electronic medical records and laboratory results, semistructured data such as Health Level7 v2 messages or Fast Healthcare Interoperability Resources, and unstructured data such as clinical notes, waveforms, and radiology images. This ensures a comprehensive view of patient information. “Variety” is about handling different types of data. It does not address data quality (the focus of “veracity”), or the usefulness of data insights generated from this comprehensive view of the patient (evaluated by “value”). While “interoperable” focuses on exchanging the same data between systems, “variety” addresses the combination of different kinds of data in one system.

#### Velocity

Velocity includes the ability to both efficiently batch process large volumes of mass data and handle speed-critical data updates in near real-time via streaming [16,17]. This duality supports time-critical medical decisions by providing up-to-date patient data and enables the rapid integration of new data sources to ensure data relevance and currency [17]. “Velocity” centers on the timeliness of data processing, independent of the amount (“volume”) or kind (“variety”) of data.

#### Veracity

Veracity highlights the critical importance of data quality and integrity, including traceability and comprehensive data management to ensure data accuracy and reliability [16]. This includes the development of a semantic understanding that allows the meaning and context of data to be captured and interpreted, which is essential for making accurate clinical decisions and maintaining patient safety [17,18]. “Veracity” emphasizes trustworthiness and data quality. This differs from “interoperable” in the FAIR principles, even though interoperability supports better veracity of data by reducing sources of error through common standards.

#### Value

Value measures the ability to query and analyze data to generate insights for research and patient care [19]. This includes support for analytics tools, such as business intelligence, big data exploration, machine learning and AI, which are essential for extracting insights from complex datasets [19]. It also considers the complexity in maintenance, handling, and management of data management tools and infrastructures to not only maximize the immediate value but also ensure the long-term sustainability and adaptability of data management solutions. “Value” focuses on the eventual benefits of data use. However, medical research has high demands on “veracity” of data as a prerequisite to create “value” from it. Furthermore, bringing different data sources with high “variety” together is often a key element of success in clinical research. Therefore, clinical “value” will often benefit from high performance in these two V’s. In addition, while the FAIR framework does not address the “value” created by data, it aims to provide the data governance necessary to create “value” from data in a sustainable way by “reuse.”

Taken together, the FAIR principles and the 5 V’s establish a robust set of both governance and technical requirements against which clinical data architectures can be systematically assessed.

### Conducting a Rapid Literature Review

**Figure 1:**
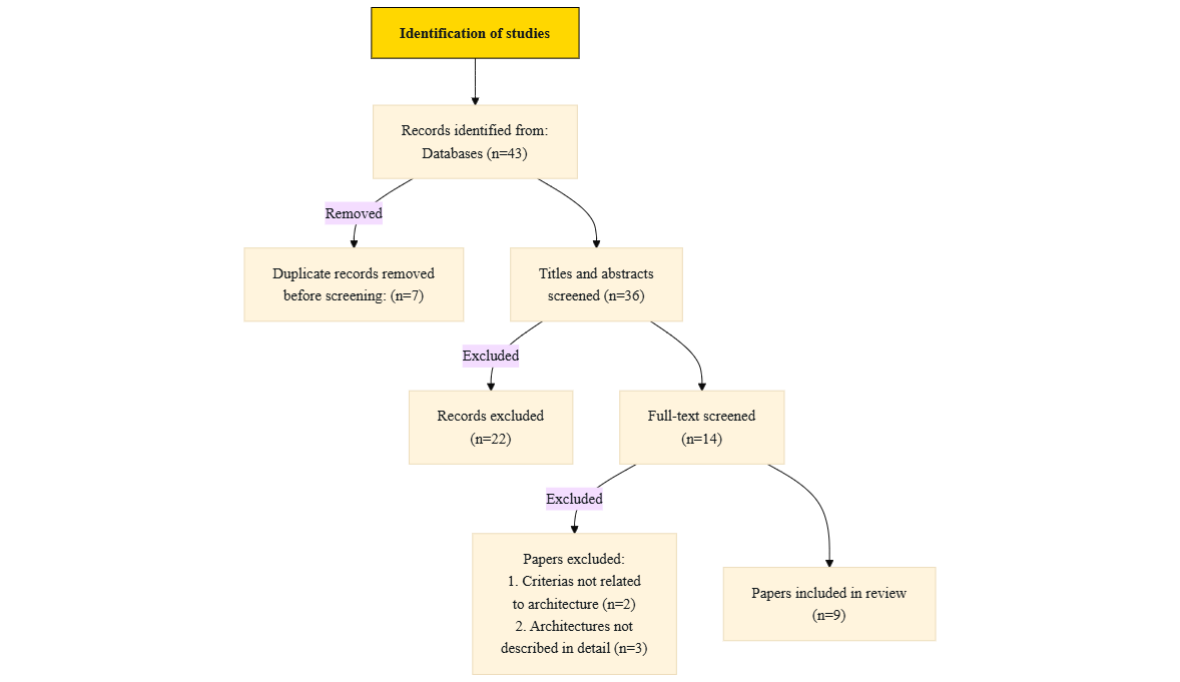
This figure illustrates the PRISMA (Preferred Reporting Items for Systematic reviews and Meta-Analyses)-like workflow used in this study. It shows the identification of records from 4 databases, the removal of duplicates, the screening of titles and abstracts, and the full-text review leading to the final inclusion of 9 articles.

Searching for data architectures combined with the FAIR principles or the 5 V’s, a total of 43 records were identified from the combined databases. After the removal of 7 duplicates, the titles and abstracts of 36 papers were screened, leading to the exclusion of 22 that did not meet the inclusion criteria. The reviewers disagreed on only 2 articles (5.6%, Cohen κ=0.88). Then, a full-text review was conducted on the remaining 14 papers, resulting in the exclusion of another 5 papers with both reviewers matching. These exclusions were primarily due to a lack of clear descriptions of data architecture or insufficient consideration of the FAIR principles or the 5 V’s of big data. The final result set contained 9 papers. This workflow is illustrated in Figure 1.

Because of the rapid literature review, the characteristics of the data management architectures (cDWHs, cDLs, and cDLHs) are shown in Multimedia Appendix 1 [8,20-27]. Although certain criteria overlap, this evaluation differentiates between governance and metadata management (FAIR) and technical performance metrics (5 V’s). This means, a quality control system that includes automated validation and detailed provenance tracking can be listed under “veracity” to show trustworthiness and consistent schema enforcement. It can also be listed under “reusability” to show its capacity to support repeated use of the data.

These results show that choosing the right data architecture for clinical research and health care depends on an institution’s needs, resources, and goals. Our literature review shows that each clinical data architecture has different requirements in terms of implementation effort, maintenance, technical expertise, and coordination. The analysis shows that the complexity increases progressively from cDWH to clinical cDL and cDLH [26,28-30].

To show the importance of these differences for AI research, we have aligned their key features with the 3 phases: design, develop, and deploy, described in the study by De Silva et al [31]. The mappings in Table 1 highlight, where each architecture supports the life cycle and where gaps remain.

**Table 1:** Comparison of the data management architectures using different stages of the artificial intelligence life cycle [31].

Life cycle stage	Clinical data warehouses	Clinical data lakes	Clinical data lakehouses
4 Data preparation	Pros: mature ETL^a^ pipelines enforce schema-on-write and ACID^b^ transactions, as well as providing fine-grained lineage, to ensure clean, well-cataloged tables, work well with relational data sources Cons: a lack of flexibility means that every new data element requires ETL reengineering; scaling up to include images, notes, or real-time feeds is costly and slow	Pros: schema-on-read allows raw files (Health Level 7 or Fast Healthcare Interoperability Resources messages, images, and waveforms) to be stored immediately, so high volumes and a wide variety of files are not a problem Cons: without disciplined metadata work, the central catalog can become weak and the data lake can degrade into a “data swamp,” which can harm later life cycle stages	Pros: it combines a lake-style raw landing with warehouse-style Delta or ACID tables, and a single metastore tracks both Cons: initial setup is complex and requires rare cloud or big data skills, so small organizations may struggle to allocate the necessary resources
5 Data exploration	Pros: fast SQL^c^ and business intelligence dashboards provide instant summaries of structured facts, such as laboratory results and drug orders Cons: unable to query unstructured data or nested JSON without significant preprocessing	Pros: notebook-driven queries can run directly on source data, such as Parquet, Optimized Row Columnar, or plain text, which enables clinicals to analyze even sensor streams or radiology notes on demand Cons: joining across modalities can be slow or error prone, quality checks must be implemented	Pros: single metadata catalog provides access to both processed and raw data regardless whether data are stored in a warehouse or an object Cons: performance tuning across lake and warehouse requires experience
7 Data preprocessing	Pros: proven ETL frameworks include validation rules, reference tables, and audit logs, which are ideal for regulated clinical features Cons: fixed schemas can hinder rapid feature engineering (eg, tokenized notes and image embeddings) and slow down iterative model work	Pros: distributed big data jobs (eg, Spark) quickly transform TB-scale raw files; researchers can test novel encodings or deep-learning pipelines quickly Cons: no built-in enforcement of data types or business rules, so imputation logic and provenance tracking must be recoded for every study	Pros: versioned open table formats allow safe roll back if a processing step fails Cons: dual workflows for big data and SQL warehouse increase the team’s long-term maintenance costs and cognitive workload

^a^ETL: extract, transform, load.

^b^ACID: atomicity, consistency, isolation, and durability.

^c^SQL: structured query language.

Stage 6 external data acquisition is the process of acquiring new data necessary to be feasible to build AI models [31]. However, once these data arrive, it will be stored in the same way as mentioned above, so the architectures enable storage and processing, but do not perform the acquisition. After stage 7, activities such as model design, training, serving, and monitoring consume the cleaned data but do not alter how it is stored. Therefore, these activities depend more on machine learning operation pipelines than choosing between cDWH, cDL or cDLH. Modern AI workflows will generate new information, for example, automatically assigned labels, capacity predictions, or new key performance indicators (KPIs). Together with additional input data, for example, through patient-reported outcome measures using electronic case report forms, these data need to be combined and harmonized with the data from clinical information systems. Besides, also provenance information and information about the generating AI workflow needs to be collected to maintain transparency and to be able to monitor risks. This process can be supported by any of these data architectures, and fits for all processes developed in research, such as new KPIs as quality metrics as well.

The following discussion examines the complexity associated with each data architecture and outlines the most appropriate use cases.

## Discussion

### Complexity

To verify and deepen these findings, we have supplemented our study with secondary literature. In the following sections, we explore 4 key aspects of complexity that are critical for health care organizations when selecting and implementing a data architecture: implementation effort, maintenance and scalability, required technical expertise, and coordination.

#### Implementation Effort

The implementation effort differs notably between the 3 architectures.

cDWHs require detailed data models, extensive extract, transform, load (ETL) processes, and a stable database system [8,32]. This usually requires a fixed project plan with defined phases for schema design, ETL development, testing, and go-live [23,32]. Although the initial effort is high, the resulting structure is clear and stable for consistent data management.

cDLs benefit from a schema-on-read approach, which reduces the need for rigid upfront data modeling. However, continuous maintenance and data harmonization demand a high level of expertise [33]. In addition, an infrastructure for big data technologies, such as Hadoop and cloud object storage, must be established. This requires experts who can set up and manage distributed systems [28,30,33]. The inclusion of streaming frameworks such as Kafka or Flume further increases the complexity [34]. In summary, the absence of a unified data model may be advantageous initially but leads to significantly more complex metadata management over time.

cDLHs’ hybrid nature combines elements from both cDWHs and cDLs. In addition to schema-on-read functionalities, cDLs require schema-on-write techniques and ACID transactions [28,34]. This dual requirement calls for the orchestration of complex ETL or extract, load, transform pipelines, robust security concepts, and distributed compute environments [28,29]. Consequently, the initial integration and development effort is very high. Often, specialized applications and frameworks are needed tools that are still in the early stages of evaluation within the medical domain.

#### Maintenance and Scalability

The maintenance and scalability of data management architectures are important factors for smooth operation.

cDWHs require continuous maintenance. Changes in data sources, schema extensions, and performance tuning demand regular attention [8,32]. Vertical scaling to increase storage and compute becomes very expensive as data volumes grow [33]. In addition, the batch-oriented structure of cDWHs may become a bottleneck when there is an increasing need for real-time data processing [23]. cDWHs offer stability and clear data governance but may struggle with the costs and limitations of scaling for real-time applications.

cDLs are maintained through active monitoring, strict data governance, and continuous data quality management. These measures help to avoid the creation of data swamps. The schema-on-read method requires constant adaptation of ingestion pipelines and greater coordination during verification [35]. In terms of scalability, the use of distributed file systems allows for affordable horizontal scaling, making it easier to handle large and diverse datasets [35]. cDLs offer flexible maintenance and cost-effective scalability but require careful coordination and robust data governance to maintain data quality.

cDLHs combine components from cDWHs and cDLs. This integration leads to more complex maintenance tasks, as both cDL and cDWH elements must be managed by multiple granular applications for distributed systems [28]. Although cDLHs support dynamic scalability for both compute and storage capacity, this flexibility requires specific expertise in resource and cost management [34]. In summary, cDLHs offer dynamic and scalable solutions that bridge the gap between structured and unstructured data, but require a more complex maintenance management.

#### Required Technical Expertise

The technical expertise needed to implement and maintain clinical data management systems is a key consideration.

cDWHs rely on relational database systems, structured query language, ETL tools, and established data warehousing concepts, including data modeling [8,22,32]. An additional technical stack and expertise is required for extended requirements, such as near real-time processing. cDWHs depend on well-established database technologies and practices, but advanced processing needs may require extra skills and tools.

cDLs require expertise in big data frameworks, such as Hadoop and Spark, cloud storage solutions, streaming tools such as Kafka or Flume, and technical expertise in distributed systems [20,35]. Users must also be skilled in analyzing and modeling unstructured data [35]. Moreover, more extensive change management and monitoring are necessary because changes are only detected when data are read from the lake. cDLs demand specialized big data skills and a proactive approach to change management, making them suitable for organizations with robust technical capabilities.

cDLHs combine traditional data warehousing with modern big data techniques. They require interdisciplinary expertise covering both classic data warehouse principles (eg, schema-on-write and ACID transactions) and contemporary big data methods (eg, schema-on-read, distributed systems, and streaming). Often, these systems are cloud-native, which means additional skills in cloud technologies and DevOps practices (such as Docker, Kubernetes, security, and identity and access management) are needed even for on-premise solutions [28,34,36]. cDLHs require a broad range of technical expertise across both traditional and modern data management technologies, posing a challenge for organizations with limited specialized resources.

#### Complexity of Coordination

cDWHs operate with a centralized governance model where responsibilities are clearly defined. Departments and IT teams must work closely to ensure continuous schema adjustments and maintain data quality within the data models [8,32,33]. cDWHs rely on largely internal coordination, as a single main instance, such as a clinic or data integration center, typically manages the system.

cDLs use a decentralized and flexible approach, which demands robust metadata strategies. Different domains, such as laboratory data, imaging, and genomics, must be coordinated effectively [33,35]. Coordination in cDLs is particularly challenging when integrating new data sources, establishing new governance standards, or maintaining schema changes [35].

cDLHs require synchronization of governance and security updates across both the cDLs and cDWHs components [29,30,36]. They have to manage the complex requirements of big data systems alongside traditional data warehousing. This hybrid processing approach, which integrates both near real-time and batch processing workflows, requires careful coordination to ensure seamless operation and data integrity cDLHs result in increased coordination complexity due to their combined systems and the need to manage parallel processing methods.

### Limitations and Additional Considerations

#### General Limitations

Our primary focus in this research was on comparing data management architectures regardless of local conditions. Still, our experience shows that factors such as cost implications and legacy system integration are critical. Furthermore, we excluded alternative data integration architectures and acknowledged the inherent limitations of frameworks, such as the FAIR principles and the 5 V’s of big data. We briefly address these aspects here to provide a broader perspective.

Some essential requirements for clinical data management are not fully covered by the FAIR principles or the 5 V’s of big data. These include privacy and confidentiality, audit and compliance mechanisms, management and documentation of ethical consent, long-term archiving, risk management, detailed user access management, and cultural sensitivity. However, it should be noted that regulatory compliance and the protection of sensitive data must always be ensured, regardless of the chosen data architecture. Therefore, we did not include these aspects in the comparison of data architectures, as they should be addressed by separate legal and organizational measures. A comprehensive data management strategy must take into account specific legal and ethical requirements in addition to the FAIR principles and the 5 V’s to ensure holistic and compliant data management.

It is important to note that this study does not consider data integration architectures such as lambda (batch) and kappa (stream). Similarly, no combination or generalization of approaches, such as data fabric or data mesh, have been evaluated for several reasons. Most importantly, data fabric was not considered a viable option because in the clinical domain, production systems cannot be accessed for complex queries. Furthermore, data mesh represents an organizational approach that focuses on domain-specific data products rather than traditional architectural archetypes, such as data lakes or data warehouses.

#### Cost Analysis and Resource Implications

In addition to technical and organizational considerations, cost analysis plays a critical role in the selection of a data management architecture. For example, while cDWHs may have high upfront costs due to extensive ETL development and schema design, they may offer lower long-term operational costs due to their stable nature. In contrast, cDLs offer cost-effective scalability for large datasets, but require ongoing investment in metadata management and system monitoring. cDLHs promise a balance between structure and flexibility, but require significant initial integration costs and ongoing maintenance. Future implementations should carefully weigh these cost implications against the performance and scalability benefits of each architecture.

#### Legacy System Integration

Integration with legacy clinical systems remains a critical factor. cDWHs generally offer smoother integration with existing relational databases and established clinical information systems. In contrast, cDLs may require additional data transformation layers and custom connectors to interface effectively with legacy systems. While cDLHs aim to combine the strengths of both, their hybrid nature can present challenges when integrating older data systems. Institutions should consider their current infrastructure and plan transitional strategies that bridge legacy systems with modern data architectures.

### Application Scenarios and Recommendations

#### Overview

Depending on the type of data, analysis requirements and available resources, a different model is recommended in each case. In the following sections, we describe the scenarios in which each of the 3 architectures offer the greatest benefits.

#### Clinical Data Warehouses

cDWHs are ideal for environments that require consistent reporting, strong compliance, and reliable analysis of structured data. They work best when data are mostly relational and fixed schemas ensure high data quality. Moreover, the structured and standardized character of cDWHs provides a stable foundation for AI applications, where reliable data are essential for training robust machine learning models. This is particularly useful for hospital governance and controlling, where clear and powerful analysis is required.

Scenarios where cDWHs should be considered:

- Structured analysis and compliance; in scenarios where data are primarily in tabular form (for instance, as recorded in a hospital information system) and there is an urgent need for rigorous data protection and comprehensive traceability, the optimal solution is often a cDWH. Stable reporting, long-term trend analyses or controlling systems as well as business intelligence tools with regular audits and fixed KPIs benefit from a cDWH.
- Resource-limited IT environments; in settings where structured query language expertise is available but there is limited capacity for complex big data or cloud solutions. Therefore, small- or medium-sized clinics may prefer a cDWH. These institutions can rely on well-established ETL processes and structured reports.
- Low to moderate data diversity; departments that record mostly numerical and tabular data (as seen in standardized clinical management systems) benefit from the fixed schema of a cDWH. The simplicity of the data model helps maintain consistency and ease of reporting and research.
- Legacy system integration; institutions with established relational databases or enterprise resource planning systems may find it easier to integrate these with a cDWH. This reduces the need for extensive data transformation and minimizes complexity.

Although cDWHs often involve high initial implementation effort, their structured nature ensures clear data governance and auditability. However, they may be less suitable for real-time processing and highly diverse data sources.

Real-world implementations: the Enterprise Clinical Research Data Warehouse at the Hannover Medical School contains data from several primary systems on more than 2.1 million distinct patients, with over 500 million data points in 48 research-data requests. It was used for epidemiological studies, cohort identification, as well as validation and data enrichment [32].

The MOSAIC i2b2 data warehouse integrates HER extracts, local health-agency records, and environmental data for type 2 diabetes data, enabling rapid cohort discovery and complex analytics [27].

This illustrates how a carefully designed schema can speed up the process of conducting AI studies that have been approved by ethics committees, while also meeting strict audit requirements.

#### Clinical Data Lake

cDLs are designed for exploratory research and the management of heterogeneous datasets. They enable organizations to store large volumes of raw data in its original format, which is useful when data arrives in many different types and formats. In addition, this flexibility is a key enabler for AI research, as it allows researchers to experiment with diverse data types and develop innovative algorithms that can learn from unstructured and semistructured information.

Scenarios where cDLs should be considered:

- Exploratory research; in projects where data requirements are not yet fully defined, a cDL offers the flexibility to store various data types. Researchers can work with unstructured data, such as doctors’ notes, images, and sensor outputs without a fixed schema.
- Machine learning prototyping; a cDL is ideal for experimental machine learning or data science projects. It allows users to quickly explore data, test different algorithms, and develop without the need for complex ETL pipelines or strict data modeling. This supports innovative research.
- Analysis of log and streaming data; when near-real-time data are needed, such as recordings from Internet of Things devices or sensor networks, a cDL can efficiently handle fast data ingestion. For example, it can support automated anomaly detection in patient vital signs or system logs.
- Cost-effective storage of high-volume data and compute; due to the big data paradigm, cDLs offer scalable, distributed storage that can handle petabytes of data. This makes them well suited for institutions collecting large amounts of diverse data on a limited budget.
- Future-proofing data; by storing raw data, a cDL allows organizations to reprocess or model the data later, as new analytic methods emerge. This is particularly valuable in fast-evolving research environments.

While cDLs offer cost-effective scalability and flexibility (via a schema-on-read approach), they demand disciplined metadata management and robust data governance. Without these measures, there is the risk of creating a data swamp.

Consequently, managing the complexity of heterogeneous data may require specialized technical expertise.

Regarding real-world implementations, the study by Gentner et al [37] analyzes several applications for data lakes in health care. They combine electronic health records with medical images and sensor, enabling exploratory data analysis on multi-source, heterogeneous data.

Al-Hgaish et al [25] created a schema-on-read data lake containing 35,840 drug entries, which were not clustered into 8568 groups using predefined models. This showcased insights from semistructured inputs.

Consequently, a cDL can scale economically, accept heterogeneous inputs while giving fast insights and processing.

#### Clinical Data Lakehouse

cDLHs combine the strengths of cDWHs and cDLs. They offer the robust governance and ACID compliance of a cDWH along with the flexibility and scalability of a cDL. Crucially, this hybrid approach supports advanced AI-driven analytics by ensuring that data remains both standardized and adaptable, which is vital for real-time processing and training of machine learning models. This hybrid approach is best suited to institutions that need both structured reporting and the capacity to manage unstructured data. However, this adds more complexity and requires high technical expertise.

Scenarios where cDLs should be considered:

- Comprehensive data integration; in large, research-intensive hospitals, a cDLH can integrate classic tabular data with vast amounts of unstructured research data (such as images and omics data). This allows the same dataset to be used for standard reporting and advanced AI applications.
- Real-time and batch processing; institutions that require both routine batch analyses and live data streaming for real-time decision support benefit from a cDLH. For example, care centers with real-time alarm systems in intensive care units can use a cDLH to support both immediate and historical analyses.
- Multi-stakeholder research platforms; when several partners (such as clinics, research institutions, and industry) collaborate, a cDLH provides a unified platform. It supports strict access controls and data governance while offering the flexibility needed for varied analytical or machine learning methods.
- Long-term investment in flexibility; organizations planning for future growth may choose a cDLH to avoid redundant systems. By integrating reporting, data science, real-time analyses, and exploratory research in one platform, they can reduce long-term operational complexity and ensure users acceptability.

The main challenge with cDLHs is the high complexity of implementation and maintenance. They require a broad range of technical expertise and resources to manage both traditional data warehousing and modern big data processes. However, for large organizations with complex data landscapes, the benefits of a unified platform can outweigh these challenges.

Regarding real-world implementations, Xiao et al’s [29] MHDML lakehouse ingests 63 GB of structured electronic health record data and 67.4 TB of unstructured imaging or waveform data for a sepsis cohort. It then reruns the same pipeline on knee osteoarthritis cases, demonstrating multimodal integration covering acute and chronic scenarios. Barnes et al [26] created a federated biomedical research hub that unifies genomics and imaging data from more than 400,000 research participants over several data sources for cancer and genetics research [26]. Begoli et al [36] report on a secure cDL at Oak Ridge National Laboratory that streams electronic health records, genomics, and administrative data into Parquet tables, containing more than 500 TB of data while satisfying the Health Insurance Portability and Accountability Act constraints. Together, these cases demonstrate how the lakehouse pattern can provide ACID controls and fine-grained access, scaling from single-hospital workloads to petabyte-scale, multipurpose resources.

### Conclusions

**Figure 2:**
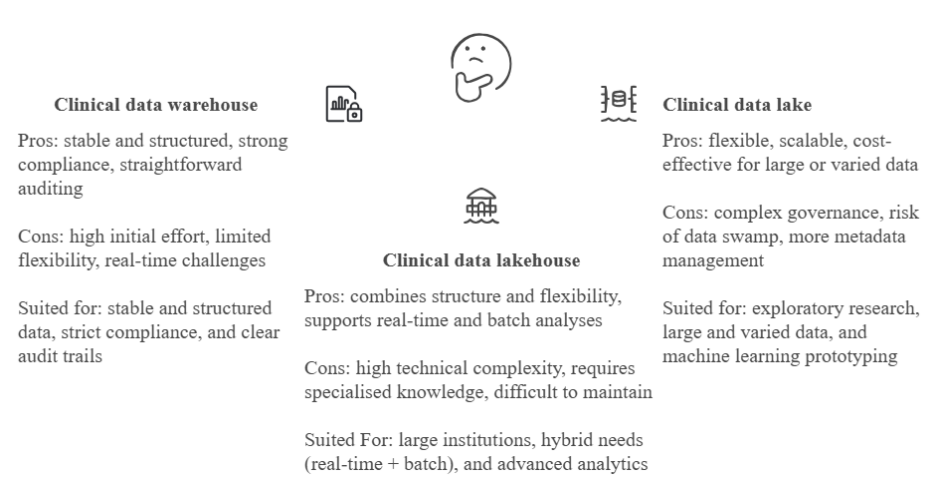
A comparative summary of clinical data warehouses, clinical data lakes, and clinical data lakehouses. It highlights the strengths and weaknesses of each architecture based on the FAIR (findable, accessible, interoperable, and reusable) principles and the big data 5 Vs (volume, variety, velocity, veracity, and value), summarizing key features, advantages, and limitations.

In summary, each data management architecture offers distinct advantages and limitations, as illustrated in Figure 2. cDWHs are best suited for stable, structured environments with strong compliance and legacy system integration. They are ideal when clear, predefined data models are needed and when audit trails are critical. cDLs provide a flexible, cost-effective solution for exploratory research and high-volume, heterogeneous datasets. They excel in environments where rapid prototyping and scalability are key. cDLHs combine the strengths of both approaches, delivering robust governance alongside flexibility. They are best suited for large, research-intensive institutions that require both real-time processing and traditional reporting.

The choice of architecture should consider immediate analytical needs, long-term scalability, technical expertise, and overall maintenance complexity. Health care organizations must balance these factors with their available resources and strategic goals.

This study emphasizes the importance of choosing the right data management architecture for medical research, patient care, and advanced AI applications. The analysis shows that cDWHs, cDLs, and cDLHs not only offer distinct strengths and weaknesses but also provide the essential foundation of reliable, standardized data that AI systems require for effective learning and decision support. The choice depends on the unique needs of each institution. By conducting this analysis, we have provided a clear framework to help health care institutions to identify the most suitable data architecture.

Using our comparison and recommendations, health care organizations can make focused decisions about which data architecture suits their needs to manage big data effectively. Focusing on the potential, cDLHs would be best suited for the diverse requirements of the health care sector, as they offer the most diverse functionalities and flexibility. However, cDLHs have the disadvantage of requiring high technical expertise and involving complex integration efforts. Future research should focus on fine-tuning cDLHs with the aim of reducing their complexity and integrating medical standards more effectively. This will expand their usability while meeting the high demands of the health care sector.

## Appendix

Multimedia Appendix 1 Comparison of the data management architectures using the FAIR (findable, accessible, interoperable, and reusable) principles and 5 Vs (volume, variety, velocity, veracity, and value) of big data.

## Abbreviations

ACID: atomicity, consistency, isolation, and durability
AI: artificial intelligence
cDL: clinical data lake
cDLH: clinical data lakehouse
cDWH: clinical data warehouse
ETL: extract, transform, load
KPI: key performance indicator
PRISMA: Preferred Reporting Items for Systematic reviews and Meta-Analyses

## References

[1] Shilo S, Rossman H, Segal E (2020). Axes of a revolution: challenges and promises of big data in healthcare. Nat Med.

[2] Baloch L, Bazai SU, Marjan S, Aftab F, Aslam S, Neo T, Amphawan A (2023). A review of big data trends and challenges in healthcare. Int J Technol.

[3] Daten - Volumen der weltweit generierten Daten bis 2028. Statista.

[4] Mendhe D, Dogra A, Nair PS, Punitha S, Preetha KS (2024). AI-enabled data-driven approaches for personalized medicine and healthcare analytics. Proceedings of the 9th International Conference on Science Technology Engineering and Mathematics.

[5] Schwabe D, Becker K, Seyferth M, Klaß A, Schaeffter T (2024). The METRIC-framework for assessing data quality for trustworthy AI in medicine: a systematic review. NPJ Digit Med.

[6] Mandl KD, Gottlieb D, Mandel JC (2024). Integration of AI in healthcare requires an interoperable digital data ecosystem. Nat Med.

[7] Pavlenko E, Strech D, Langhof H (2020). Implementation of data access and use procedures in clinical data warehouses. A systematic review of literature and publicly available policies. BMC Med Inform Decis Mak.

[8] Sebaa A, Chikh F, Nouicer A, Tari A (2018). Medical big data warehouse: architecture and system design, a case study: improving healthcare resources distribution. J Med Syst.

[9] Babu H, Arivazhagn D, Kumar G, Patil S, Akhtar M, Bakar E (2023). Examining the potential benefits and challenges of utilizing AI for big data analysis. Proceedings of the 3rd International Conference on Smart Generation Computing, Communication and Networking.

[10] Mungoli N Scalable, distributed AI frameworks: leveraging cloud computing for enhanced deep learning performance and efficiency. arXiv. Preprint posted online on April 26, 2023.

[11] El Aissi, ME, Benjelloun S, Loukili Y, Lakhrissi Y, El Boushaki AE, Chougrad H, Ali SE (2020). Data lake versus data warehouse architecture: a comparative study. Proceedings of the 6th International Conference on Wireless Technologies, Embedded, and Intelligent Systems.

[12] Zaharia M, Ghodsi A, Xin R, Armbrust M (2021). Lakehouse: a new generation of open platforms that unify data warehousing and advanced analytics. Proceedings of the 11th Conference on Innovative Data Systems Research.

[13] Sinaci AA, Núñez-Benjumea FJ, Gencturk M, Jauer Ml, Deserno T, Chronaki C, Cangioli G, Cavero-Barca C, Rodríguez-Pérez JM, Pérez-Pérez MM, Laleci Erturkmen GB, Hernández-Pérez T, Méndez-Rodríguez E, Parra-Calderón CL (2020). From raw data to FAIR data: the FAIRification workflow for health research. Methods Inf Med.

[14] Holub P, Kohlmayer F, Prasser F, Mayrhofer MT, Schlünder I, Martin GM, Casati S, Koumakis L, Wutte A, Strapagiel D, Anton G, Zanetti G, Sezerman OU, Mendy M, Valík D, Lavitrano M, Dagher G, Zatloukal K, van Ommen GB, Litton J, Kozera (2018). Enhancing reuse of data and biological material in medical research: from FAIR to FAIR-health. Biopreserv Biobank.

[15] Wilkinson MD, Dumontier M, Aalbersberg IJ, Appleton G, Axton M, Baak A, Blomberg N, Boiten J, da Silva Santos LB, Bourne PE, Bouwman J, Brookes AJ, Clark T, Crosas M, Dillo I, Dumon O, Edmunds S, Evelo CT, Finkers R, Gonzalez-Beltran A, Gray AJ, Groth P, Goble C, Grethe JS, Heringa J, 't Hoen PA, Hooft R, Kuhn T, Kok R, Kok J, Lusher SJ, Martone ME, Mons A, Packer AL, Persson B, Rocca-Serra P, Roos M, van Schaik R, Sansone S, Schultes E, Sengstag T, Slater T, Strawn G, Swertz MA, Thompson M, van der Lei J, van Mulligen E, Velterop J, Waagmeester A, Wittenburg P, Wolstencroft K, Zhao J, Mons B (2016). The FAIR Guiding Principles for scientific data management and stewardship. Sci Data.

[16] Bellazzi R (2014). Big data and biomedical informatics: a challenging opportunity. Yearb Med Inform.

[17] Mooney SJ, Westreich DJ, El-Sayed AM (2015). Commentary: epidemiology in the era of big data. Epidemiology.

[18] Lee CH, Yoon HJ (2017). Medical big data: promise and challenges. Kidney Res Clin Pract.

[19] Ting DS, Deshmukh R, Ting DS, Ang M (2023). Big data in corneal diseases and cataract: current applications and future directions. Front Big Data.

[20] Natarajan K, Weng C, Sengupta S (2023). A model for multi-institutional clinical data repository. Stud Health Technol Inform.

[21] Bellandi V, Ceravolo P, Maggesi J, Maghool S (2024). Data management for continuous learning in EHR systems. ACM Trans Internet Technol (Forthcoming).

[22] Parciak M, Suhr M, Schmidt C, Bönisch C, Löhnhardt B, Kesztyüs D, Kesztyüs T (2023). FAIRness through automation: development of an automated medical data integration infrastructure for FAIR health data in a maximum care university hospital. BMC Med Inform Decis Mak.

[23] Lacey Jr JV, Chung NT, Hughes P, Benbow JL, Duffy C, Savage KE, Spielfogel ES, Wang SS, Martinez ME, Chandra S (2020). Insights from adopting a data commons approach for large-scale observational cohort studies: the California teachers study. Cancer Epidemiol Biomarkers Prev.

[24] Guilbert B, Rieu T, Delamarre D, Laurent F, Serre F, Leroy S, Paillard E, Le Buhan N, Ribault A, Cuggia M (2024). ONCO-FAIR project: improving data interoperability in oncology chemotherapy treatments for data reuse. Stud Health Technol Inform.

[25] AL-Hgaish A, ALzyadat W, Al-Fayoumi M, Alhroob AM, Thunibat A (2019). Preserve quality medical drug data toward meaningful data lake by cluster. Int J Eng Technol.

[26] Barnes C, Bajracharya B, Cannalte M, Gowani Z, Haley W, Kass-Hout T, Hernandez K, Ingram M, Juvvala HP, Kuffel G, Martinov P, Maxwell JM, McCann J, Malhotra A, Metoki-Shlubsky N, Meyer C, Paredes A, Qureshi J, Ritter X, Schumm P, Shao M, Sheth U, Simmons T, VanTol A, Zhang Z, Grossman RL (2022). The Biomedical Research Hub: a federated platform for patient research data. J Am Med Inform Assoc.

[27] Dhayne H, Haque R, Kilany R, Taher Y (2019). In search of big medical data integration solutions - a comprehensive survey. IEEE Access.

[28] Harby AA, Zulkernine F (2022). From data warehouse to Lakehouse: a comparative review. Proceedings of the 2022 IEEE International Conference on Big Data.

[29] Xiao Q, Zheng W, Mao C, Hou W, Lan H, Han D, Duan Y, Ren P, Sheng M (2022). MHDML: construction of a medical Lakehouse for multi-source heterogeneous data. Proceedings of the 11th International Conference on Health Information Science.

[30] Čuš B, Golec D (2023). Data Lakehouse: benefits in small and medium enterprises. J Innov Bus Manag.

[31] De Silva D, Alahakoon D (2022). An artificial intelligence life cycle: from conception to production. Patterns (N Y).

[32] Gerbel S, Laser H, Schönfeld N, Rassmann T (2019). The Hannover medical school enterprise clinical research data warehouse: 5 years of experience. Proceedings of the 13th International Conference on Data Integration in the Life Sciences.

[33] Nambiar A, Mundra D (2022). An overview of data warehouse and data lake in modern enterprise data management. Big Data Cogn Comput.

[34] Oreščanin D, Hlupić T (2021). Data Lakehouse - a novel step in analytics architecture. Proceedings of the 44th International Convention on Information, Communication and Electronic Technology.

[35] Quix C, Hai R, Sakr S, Zomaya AY (2018). Data lake. Encyclopedia of Big Data Technologies.

[36] Begoli E, Goethert I, Knight K (2021). A Lakehouse architecture for the management and analysis of heterogeneous data for biomedical research and mega-biobanks. Proceedings of the 2021 IEEE International Conference on Big Data.

[37] Gentner T, Neitzel T, Schulze J, Gerschner F, Theissler A (2023). Data lakes in healthcare: applications and benefits from the perspective of data sources and players. Procedia Comput Sci.

