# Supplementary material for: Enhancing Clinical Data Infrastructure for AI Research: Comparative Evaluation of Data Management Architectures
Source: J Med Internet Res. 2025 Aug 1;27:e74976. doi: 10.2196/74976 (PMC12357119; doi:10.2196/74976)
Supplement: Multimedia Appendix 1 [file jmir_v27i1e74976_app1.docx]

**Multimedia Appendix 1.** Comparison of the data management architectures using the FAIR principles and 5 Vs of big data [8,20-27].

| Requirement | cDWH [8,20,22-24,26,27] | cDL [20,22,25-27] | cDLH [21,26] |
| --- | --- | --- | --- |
| Findable | Pros: centralized metadata with unique persistent IDs, standardized schemas, integrated catalogs, and detailed data lineage | Pros: flexible schema-on-read enables dynamic cataloging, supports diverse data formats | Pros: hybrid metadata management combines centralized and federated approaches, global unique identifiers, standardized API^a^ interfaces enhance integration, supports structured and unstructured data |
|  | Cons: fixed structure limits adaptation to heterogeneous data, high maintenance effort, scalability challenges | Cons: decentralized metadata can lead to inconsistencies, missing fixed data schema complicates cross-system retrieval, requires continuous harmonization | Cons: Cons: high initial financial and resource investment (including hardware acquisition, infrastructure setup, and extensive personnel training), coupled with complex interorganizational coordination |
| Accessible | Pros: centralized repository, standardized SQL^b^ access, robust authorization controls, high data availability, user-friendly interfaces | Pros: flexible REST^c^ API access, support for Health Level 7 and Fast Healthcare Interoperability Resources data feeds, diverse access methods, versioned data access | Pros: open and standardized APIs and interfaces (REST and SQL), scalability through distributed services, high availability, seamless integration of structured and unstructured data |
|  | Cons: limited flexibility for external access, expensive scaling for very large volumes | Cons: variable performance under heavy loads, requires advanced technical skills, complex protocol management | Cons: complex initial setup for multi-layered data access, requiring advanced technical expertise in configuring distributed systems, securing diverse interfaces (eg, REST and SQL), and integrating heterogeneous APIs |
| Interoperable | Pros: standardized SQL‐based data models, centralized extract, transform, load ensuring traceable data harmonization, comprehensive metadata management | Pros: supports semantic focus on data governance through metadata, flexible integration with external systems, flexible schema‐on‐read | Pros: unified architecture leveraging shared services, controlled vocabularies with standardized API frameworks, hybrid real-time and batch processing, integrated metadata frameworks |
|  | Cons: rigid, batch‐oriented schema limits rapid updates, high curation and maintenance effort | Cons: decentralized structure complicates harmonization, continuous integration demands, potential lack of atomicity, consistency, isolation, and durability consistency | Cons: significant initial integration and governance effort, coordination challenges across federated systems |
| Reusable | Pros: structured data model, detailed provenance, comprehensive metadata, robust quality controls, strong compliance | Pros: flexible schema-on-read, accommodates diverse data types, supports curated data marts, enables versioning | Pros: combines structured reliability with flexible data handling, semantic enrichment, integrated metadata and versioning |
|  | Cons: fixed schemas limit adaptability and exploration of new research questions | Cons: inconsistent metadata, variable data quality | Cons: integration and maintenance require significant resources |
| Volume | Pros: optimized for large volumes of structured data with efficient indexing and query performance | Pros: highly scalable distributed storage is cost-effective for very large (petabyte-scale) raw data | Pros: distributed software architecture supports dynamic scaling, balanced support for structured and unstructured data with large volumes |
|  | Cons: requires expensive vertical scaling, relies on batch processing causing performance peaks |  | Cons: complex resource management and integration required |
| Variety | Pros: defined schemas maintain consistent quality for homogeneous data types from multiple data sources | Pros: schema-on-read easily integrates structured, semistructured, and unstructured data; highly scalable for diverse formats | Pros: supports diverse data types and evolving standards, supports multiple data models |
|  | Cons: inflexible when facing rapidly changing or unstructured formats, integration of heterogeneous data types requires specialized solutions or blobs | Cons: requires robust ontology mapping to maintain semantic consistency | Cons: demands ongoing monitoring, requires strong governance to manage new formats effectively |
| Velocity | Pros: established batch processing, consistent scheduled updates, ideal for retrospective analysis | Pros: near real-time ingestion, rapid processing enabling timely analytics, support for continuous data streams | Pros: integrates real-time and batch processing using advanced frameworks (eg, Delta Lake), achieves low-latency pipelines |
|  | Cons: limited real-time capabilities, higher latency when current data are needed, scalability challenges | Cons: complex resource optimization, increased integration and harmonization demands | Cons: challenging configuration and scaling |
| Veracity | Pros: strong data quality management, harmonized data, and clear lineage build trust | Pros: retains original integrity of data, versioning supports traceability | Atomicity, consistency, isolation, and durability transactions; enforced schemas and advanced version management ensure high data integrity and real-time validation |
|  | Cons: complex handling of unstructured data, basic versioning and error correction restrict comprehensive quality assurance | Cons: complex harmonization of heterogeneous data and limited lineage tracking can challenge consistency | Cons: demands higher technical and governance efforts to maintain quality |
| Value | Pros: centralized integration produces reliable research utilities, analytics, and reporting | Pros: enables flexible data exploration with real-time insights, supports diverse data types and exploratory research | Pros: combines reliability of traditional warehousing with innovative data handling, high data quality, interoperability and scalability add overall value |
|  | Cons: high implementation and maintenance costs, limited flexibility may restrict secondary use | Cons: continuous management required to ensure long-term value | Cons: Specialist expertise and significant integration efforts needed for effective management |

^a^API: application programming interface.

^b^SQL: structured query language.

^c^REST: representational state transfer.

|  |  |  |  |
| --- | --- | --- | --- |
|  |  |  |  |
|  |  |  |  |
|  |  |  |  |
|  |  |  |  |
|  |  |  |  |
|  |  |  |  |
|  |  |  |  |
|  |  |  |  |
|  |  |  |  |
